# Integrated Experimental–Computational Framework for Drug Transport Quantification in 3D *Microtissues*™

**DOI:** 10.3390/mi17030332

**Published:** 2026-03-09

**Authors:** Ramisa Fariha, Jad Hamze, Oluwanifemi David Okoh, Emma Rothkopf, Anubhav Tripathi

**Affiliations:** 1 Center for Biomedical Engineering, School of Engineering, Brown University, 182 Hope Street, Providence, RI 02912, USA; 2 Department of Biology and Medicine, Brown University, Providence, RI 02912, USA

**Keywords:** 3D microtissues, microphysiological systems, drug absorption, tissue engineering, LC–MS/MS, in vitro-to-in vivo extrapolation, precision medicine

## Abstract

While traditional 2D in vitro models have been widely used for drug screening, 3D tissue culture systems are gaining traction due to their superior ability to replicate in vivo tumor microenvironments. In this study, we utilize *Microtissues*™, a validated, scaffold-free, high-throughput 3D tissue culture platform, as the basis for a microscale tissue-engineered model to study drug absorption and transport dynamics. Despite their physiological relevance, such 3D constructs pose analytical challenges, particularly in quantifying trace drug levels within the microenvironment. We developed and validated an integrated experimental workflow combining optimized liquid–liquid extraction and protein precipitation with LC-MS/MS analysis to accurately quantify paclitaxel absorption in *Microtissues*™ molds using small sample volumes. The assay achieved a validated lower limit of quantification of 0.03 μM, with robust linearity across analytical runs (R^2^ ≥ 0.90; best-run performance > 0.99) and precision (CV ≤ 10%) across both MRMs. This microengineered in vitro system allows for precise characterization of drug–tissue interactions in MCF7 breast cancer *Microtissues*™, enabling in vitro-to-in vivo extrapolation (IVIVE) relevant to therapeutic optimization. The platform’s scalability and modularity support its application in precision medicine, where patient-derived microtissues can guide individualized treatment decisions.

## 1. Introduction

Three-dimensional (3D) microtissue and spheroid models have emerged as powerful microphysiological systems (MPSs) for studying cancer biology, therapeutic response, and drug transport phenomena [1,2,3,4] Unlike traditional 2D monolayers, 3D microtissues better recapitulate the structural and biochemical features of the tumor microenvironment, including cell–cell contacts, extracellular matrix (ECM) components, oxygen gradients, and spatially heterogeneous metabolism [5,6,7]. These features influence drug penetration, retention, and efficacy, making microengineered platforms increasingly important for preclinical evaluation of chemotherapeutic agents [3,8].

Breast cancer, in particular, remains a major global health concern and is the second leading cause of cancer-related deaths in women [9]. It accounts for approximately 30% of newly diagnosed cancers in women annually, highlighting the need for more predictive preclinical testing models [9,10]. While 2D models have been foundational in cancer research, they fail to represent in vivo tumor complexity, prompting a transition to 3D systems [1,11,12]. Supplemental Table S1 outlines the key differences between 2D and 3D culture platforms [12,13,14]. Paclitaxel (PTX), a widely used anticancer drug, exhibits complex transport behavior due to its high hydrophobicity, extensive protein binding, poor aqueous solubility, and strong affinity for intracellular structures [15,16,17]. As a result, the nominal drug concentration in media often poorly reflects actual intracellular exposure, especially in 3D culture systems where diffusion barriers and sequestration phenomena are amplified [3,18,19]. Quantifying how drugs partition into, accumulate within, and diffuse across 3D constructs is, therefore, critical for interpreting cytotoxicity data, benchmark dosing, and in vitro-to-in vivo extrapolation (IVIVE) [6,20,21].

*Microtissues*™ is a scaffold-free, self-assembling 3D culture system that enables the reproducible formation of microphysiological tissue constructs, serving as an engineered environment for studying drug transport, viability, and in vitro-to-in vivo extrapolation. Despite the widespread adoption of 3D culture models for drug screening, standardized methods for direct measurement of drug uptake within *Microtissues*™ remain lacking [3,6,8,22,23]. Most studies rely on endpoint viability assays (e.g., ATP or live–dead staining), which report phenotypic outcomes but not actual pharmacokinetic exposure [7,24,25]. This disconnect creates uncertainty about whether observed biological effects stem from true differences in drug potency or from microenvironment-dependent transport limitations [11,13,21,26]. Supplementary Table S2 compares *Microtissues*™ to other commonly used 3D culture platforms, underscoring the need for analytical tools that can resolve these functional differences. Liquid chromatography–tandem mass spectrometry (LC-MS/MS) is a sensitive and specific technique capable of quantifying low concentrations of drugs in complex biological matrices [27,28,29,30,31,32,33,34]. While widely used in clinical pharmacology and plasma metabolite analysis, its integration with engineered 3D in vitro platforms remains limited [14,28]. Coupling LC-MS/MS with computational modeling provides a powerful toolkit to characterize drug diffusion, accumulation, and spatial gradients within 3D tissues [25]. These integrative approaches are essential for developing predictive and reproducible microengineered systems for drug transport studies [5,10,35].

In this study, we present a validated LC-MS/MS-based analytical framework, integrated with engineered 3D *Microtissues*™ and computational modeling, to quantitatively assess drug absorption, retention, and time-dependent transport in microscale in vitro systems. PTX uptake was evaluated over 24 and extended 60 h exposure periods at concentrations ranging from 0.05 μM to 5 μM, with intracellular drug levels directly quantified using an optimized extraction-based LC-MS/MS workflow. A complementary finite-element diffusion–reaction model was employed to provide a mechanistic context for transport and partitioning behavior during sample preparation, supporting interpretation of the experimental measurements.

Our findings demonstrate that:

- 3D microtissues generate microenvironment-dependent drug concentration gradients arising from platform-specific absorption and diffusion effects;
- Nominal media dosing does not reliably reflect true intracellular drug exposure in 3D microtissue systems;
- Intracellular drug accumulation exhibits nonlinear dependence on both exposure time and applied concentration;
- Quantitative drug measurement is essential for supporting reliable in vitro-to-in vivo extrapolation (IVIVE) in engineered microtissue platforms.

Together, these results establish a robust, quantitative framework for biofabricated microtissue-based drug transport analysis, enhancing the interpretability and predictive power of 3D cancer drug screening assays and supporting future precision medicine applications.

## 2. Experimental Section

### 2.1. Chemicals and Reagents

All solvents used were LC-MS grade, Baker-Analyzed (Radnor, PA, USA), with a purity >99.9% (obtained from VWR, Radnor, PA, USA), including water, acetonitrile, and DMSO used in the preparation of calibrators and operation of the LC-MS/MS. Reagent-grade formic acid (96% pure) was purchased from Fisher Scientific (Waltham, MA, USA). Paclitaxel (PTX) (10 mM in DMSO) was obtained from AdooQ Bioscience (Irvine, CA, USA), and Docetaxel (DOC) (10 mM in DMSO) was obtained from MedChem Express (Monmouth Junction, NJ, USA). Dulbecco’s Modified Eagle Medium (DMEM), penicillin/streptomycin, 1× PBS, and Fetal Bovine Serum were Gibco-Manufactured (Waltham, MA, USA) and purchased from ThermoFisher Scientific (Waltham, MA, USA). Cell-culture-grade agarose was purchased from Sigma Aldrich (St. Louis, MO, USA), and microtissue molds were purchased from *Microtissues* Inc. (Providence, RI, USA).

### 2.2. Cell Culture Conditions

*Microtissues*™ were created using 2% agarose solution (in 1× PBS), on reusable 24–96 small spheroid micromolds and equilibrated in serum-free DMEM (with L-glutamine, phenol red, and sodium pyruvate) supplemented with 1% penicillin/streptomycin for at least 24 h prior to all experiments. Human epithelial MCF7 breast cancer cells were obtained from American Tissue Culture Collection (ATCC, Rockville, MD, USA), and cultured using DMEM with L-glutamine, phenol red, and sodium pyruvate, supplemented with 10% FBS and 1% penicillin/streptomycin at 5% CO_2_ and 37 °C. Cells were expanded (P8-10) using a standard Trypsin protocol: one Versene solution wash was applied, followed by three 1× PBS washes, and ~0.05% trypsin in PBS for 5 min; they were then quenched with serum-containing media and centrifuged at 125 g for 5 min, prior to resuspension and counting. Cells were contained in 2D culture flasks at no more than 1.2 million cells per well, and the spheroids were seeded at 500 cells per spheroid, i.e., $4.8\times 10^{4}$ cells per well with media exchanged every day at DIV = 3 prior to PTX exposure. PTX solutions were made using DMEM with L-glutamine, phenol red, and sodium pyruvate, supplemented with 10% FBS and 1% penicillin/streptomycin media (also referred to as *Complete Media* hereafter) in varying concentrations (as described throughout the study). The control samples had a DMSO concentration <0.05% for consistency in all the samples across the board.

### 2.3. Drug Treatment Protocol and Empty Microtissue Study

In tandem with spheroid culture, *Microtissues*™ were made and plated, and taken through the same equilibration process as described above. Once equilibrated, these *Microtissues*™ were not seeded with cells, but rather exposed to PTX solutions over 6, 12, 24, and 60 h. The same drug solution prepared to treat the empty *Microtissues*™ was placed on empty culture wells (within the same plate) and analyzed alongside the empty *Microtissues*™ samples. Additionally, all prepared drug solutions were run on the LC-MS/MS each time to determine the true starting concentration for each experiment for future data normalization.

### 2.4. LC-MS/MS Conditions and Parameters

PTX and DOC (as internal standard) were diluted 250-fold in HPLC-grade acetonitrile and infused directly into the MS to quantify the parent mass and quantifier fragment masses. The infusion was performed using a Harvard apparatus at a flow rate of 30 μL/min. All compound transition data, together with the Entrance Voltage (EV), Collision Cell Energy (CC), and Collision Cell Lens 2 (CCL2) values, were obtained by Multiple Reaction Monitoring (MRM) optimization. Upon completing the MRM optimization, the nebulizer gas pressure, source temperature, HSID (hot surface-induced desolvation) temperature, and drying gas pressure were also optimized to obtain the best relative intensity for both MRMs of the analytes and the internal standards. After the Q1 and Q3 masses were detected, the EV, CC, and CCL2 values were re-optimized for the best signal of the analyte quantifier, qualifier, and internal standard, as listed in Table 1a,b. The probe positions were also adjusted to obtain the best signal intensity and sensitivity. The system used was a QSight 220 CR Triple Quadrupole Mass Spectrometer with LX50 HPLC front (Revvity Inc., Waltham, MA, USA), equipped with an Electron Spray Injection (ESI) source in positive ion mode. The optimized MS parameters were a nebulizer pressure of 80 psi, electrospray voltage of 5850 volts, and a source temperature of 425 °C. The drying gas was set to 60, and HSID was set to 220 °C. An Agilent Zorbax RRHD C-18 column (1.8 µm, 2.1 × 50 mm), set at 25 °C with mobile phase A (water with 0.1% Formic Acid) and mobile phase B (acetonitrile with 0.1% formic acid), was used. The final LC gradient profile was designed for a high sensitivity and a low flow rate (0.4 mL/min linear flow), with a 3 min run time per sample (Table 1a,b).

The sample tray holder was maintained at 4 °C. The autosampler needle was put through weak–strong–weak solvent wash cycles between each injection, with 70:30 = water:acetonitrile solution as the weak solvent and 100% HPLC acetonitrile as the strong solvent at a 250 µL volume for both solvents. The solvent delivery module was connected to a 10 µL needle and a 20 µL loop, with a solvent injection volume of 10 µL, 2 mm from the vial bottom.

### 2.5. Preparation of Calibrators, Quality Controls, and Extraction Solvent

The stock PTX (10 mM) was diluted through a two-step dilution process to yield a 6-point linearity series and quality controls (QC—low; QC—high) in *Complete Media* (described above). Briefly, 15 mL of *Complete Media* was aliquoted into individual glass vials with stir bars, and diluted PTX solutions were added to each vial and stirred for about 15 min at room temperature prior to overnight rocking at 4 °C. All working volumes were greater than 10 μL to avoid small-volume pipetting errors, and DMSO was normalized by volume across all calibrators, quality controls, and L0 (*Complete Media* only, with DMSO). The *Extraction Solvent* was designed for the best layer separation and PTX adhesion, in addition to maintaining a pH suitable for the column. The final *Extraction Solvent* consisted of acidified acetonitrile (to be used ice cold) (0.1% formic acid) with DOC diluted 10,000-fold, which was integrated by stirring at room temperature for at least 30 min.

The entirety of the study rendered four iterations of the linearity series, and each series was individually quantified in bulk prior to its use for any experimentation. Additionally, all the calibrators after bulk production were aliquoted in 2 mL microfuge tubes for single use (200 μL per level per experiment) and stored at −20 °C. Samples for freeze–thaw analysis were stored in larger volumes (1600 μL), and samples for stability analysis were stored separately under different temperature conditions.

### 2.6. Sample Preparation

The sample preparation optimization took place in several stages. The finalized protocol used a liquid–liquid separation technique between the *Complete Media* and the *Extraction Solvent*, which was mixed in a 1:2 ratio, vortex-mixed, and centrifuged at 0 °C for 10 min at 4600 rpm. The final supernatant collected was from the top layer, carefully avoiding the DMSO micropartition that separated the phenol red and FBS serum proteins from PTX and the organic solvent. The final extract was placed in Thermo Fisher 2 mL screw-top autosampler vials for analysis.

### 2.7. COMSOL Multiphysics Modeling

A time-dependent finite element model was developed in COMSOL Multiphysics^®^ (version 6.0) using the Transport of Diluted Species physics interface to simulate PTX partitioning across a three-phase liquid–liquid extraction system. The goal was to evaluate PTX transport behavior from complete media (aqueous, bottom-layer media), across an intermediate DMSO interface, into the organic *Extraction Solvent* (top layer), over a 10 min interval encompassing both mixing and phase settling, which is essential to optimize the sample preparation workflow.

#### 2.7.1. Model Geometry and Phases

The simulation domain was constructed as a 3-layer vertical stack representing:

- Bottom layer: *Complete Media* containing initial PTX (10 µM);
- Middle layer: DMSO interfacial region (approximately 1 mm in thickness);
- Top layer: Organic *Extraction Solvent*.

Each phase was modeled as a separate domain with defined thickness based on experimental measurements, except the DMSO layer. The DMSO was visually infinitesimally small during experimentation. The simulation domain was 3D, with axisymmetric geometry used for computational efficiency, and the mesh size was ‘fine’.

#### 2.7.2. Initial and Boundary Conditions

- Initial condition: PTX was initially confined to the *Complete Media* layer at a concentration of 10 µM; the DMSO and solvent layers were assumed drug-free at t=0.
- Interface conditions: Partitioning across the DMSO layer was modeled via continuity of flux, with no resistance imposed at interfaces.
- External boundaries: The top and side walls were set as insulating (no flux), while gravity-driven separation was not explicitly modeled (static layers only).

#### 2.7.3. Time-Dependent Simulation

The model was run for 10 min (600 s) using an implicit solver with adaptive time-stepping. Outputs were generated at specific time points: t=0, 1, and 10 min to match experimental mixing and separation time scales.

The results illustrated the progressive migration of PTX from the aqueous phase into the organic solvent through the DMSO interfacial region. The model visually captured the concentration gradient shift and transport flux, supporting the mechanistic hypothesis of interface-mediated PTX enrichment in the extraction phase.

### 2.8. Validation Against Immunoassay

To assess cellular viability, a live–dead cytotoxicity kit for mammalian cells (Thermo Fisher) was used. The kit contained 4 mM Calcein-AM (for viability) and 2 mM ethidium homodimer-1 (for cell death). The tissues (with MCF7 spheroids growing in them) were washed three times in serum-free media, with ten minutes of incubation between each wash. Once the washes were done, the manufacturer’s protocol was adjusted and optimized for the 3D spheroids and used for the assay. The wells were imaged using the Zeiss Axio Observer Z1 (ZEISS, Oberkochen, Germany) connected to an Xcite (Pittsburgh, PA, USA) fluorescence lamp (series 120 Q). The controls used were *Complete Media* for live staining and 70% ethanol for dead staining, and the EGFP (green) and dsRed (dead) channel exposures were normalized to the controls for the experimental groups.

### 2.9. DoE and Data Analysis

For the entire study, the Design of Experiment (DoE) was performed using JMP Pro 16 to optimize the number of samples to be tested for statistical significance (α = 0.05) undermultiple conditions (number of samples per round of drug exposure, number of samples for assay validation, etc.). All LC-MS/MS data were collected and analyzed using Simplicity 3Q (version 3.0) software for QSight. The data exported was further analyzed using Microsoft Excel (2024), JMP Pro 16, and GraphPad Prism (version 9.0.2) for visualization and statistical analysis.

## 3. Results and Discussion

### 3.1. Assay Development and Microscale Drug Transport Quantification

Prior to initiating cell culture experiments, it was necessary to develop and optimize a liquid chromatography–tandem mass spectrometry (LC-MS/MS) assay capable of accurate and reproducible quantification of PTX from the cell culture supernatant across multiple runs. Method development began with the evaluation of mobile-phase compositions. In two independent trials, HPLC-grade water with 0.1% formic acid was used as mobile phase A, while two candidates were tested as mobile phase B: acetonitrile with 0.1% formic acid and methanol with 0.1% formic acid.

Acetonitrile consistently produced greater signal intensities and more defined chromatographic peaks and was, therefore, selected as the organic modifier for all subsequent analyses. PTX was further evaluated in varying proportions of organic solvent (5% to 95% acetonitrile or methanol) to identify the optimal ratio for achieving high-signal quality and peak resolution. A 2:1 acetonitrile:water mixture provided robust, reproducible, bell-shaped chromatograms without compromising peak intensity.

To enable internal normalization, DOC was evaluated as an internal standard (IS) across a range of concentrations. An optimal working concentration of 0.001 nM DOC in acidified acetonitrile was selected to minimize background noise and eliminate false-positive peaks. The choice of acidified acetonitrile was further supported by the literature, indicating that PTX is unstable in alkaline and methanolic environments [36,37], especially during extraction and temperature-dependent centrifugation steps.

**Figure 1:**
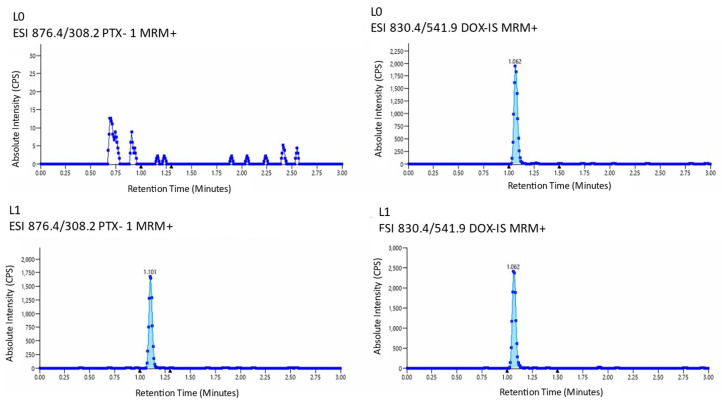
Representative chromatograms from LC–MS/MS analysis. (**Top**) Blank media (L0) containing only the internal standard (DOC) showed no detectable peak for PTX, confirming the specificity of the method. A clear DOC peak was observed, as expected. (**Bottom**) The lowest calibrator level (L1; 0.03 μM PTX) demonstrated a distinct and quantifiable PTX peak alongside the DOC internal standard, confirming sensitivity and linearity at low concentrations.

The final method produced distinct, interference-free chromatograms, as demonstrated in Figure 1, which shows representative traces for a blank matrix (media with only IS) and the lowest calibrator level (L1).

### 3.2. Partitioning Behavior and Computational Modeling of Interfacial Transport

The extraction method was designed based on the established literature describing the solubility characteristics of PTX in various solvents, and the influence of temperature and pH on its stability [16,17,38,39]. In this system, PTX was initially dissolved in dimethyl sulfoxide (DMSO) and introduced into a biphasic extraction setup consisting of *Complete Media* (aqueous phase) and an organic solvent (extraction phase). While DMSO was not initially considered as a distinct phase, centrifugation revealed a visible interfacial layer, prompting further investigation.

To model partitioning behavior, an initial two-phase distribution coefficient ($K_{D}$) was calculated using Equation (1), where $\left[A{]}_{1}\right.$ and $\left[A{]}_{2}\right.$ represent the PTX concentrations in the organic and aqueous phases, respectively [40]:

(1) $$K_{D}=\frac{{\left[A\right]}_{1}}{[{A]}_{2}}$$

This simplified model assumes direct partitioning between two bulk phases. However, the presence of the DMSO layer (evident by its distinct separation and phenol red staining) necessitated a refined, thermodynamically grounded approach.

A three-phase model was subsequently implemented, incorporating the DMSO interfacial layer as a mediator in the transfer of PTX from the aqueous phase to the organic solvent. Following interfacial thermodynamics, Equation (2) was used to describe the migration of PTX across the interface, based on the assumption that the chemical potential of PTX at the *Extraction Solvent* interface and the DMSO surface is equal [41]:

(2) $$\omega =exp[\frac{\gamma_{extraction\;solvent}^{lv}\;-\;\;\gamma_{DMSO}^{lv}}{k_{B}T}]\bar{A}$$

where

- $\gamma^{lv}$ represents the liquid–vapor surface tension of each phase;
- $k_{B}$ is the Boltzmann constant;
- T is absolute temperature (in Kelvin);
- $\bar{A}$ is the area per molecule, assumed to be approximately equal across phases.

**Figure 2:**
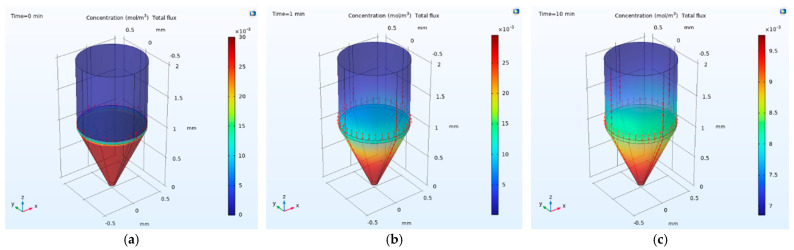
Finite element simulation of PTX partitioning across a three-phase system, representing complete media (aqueous, bottom), the DMSO interfacial layer (middle), and organic *Extraction Solvent* (top). Simulations were performed using COMSOL Multiphysics (the Transport of Diluted Species module) to visualize time-dependent drug migration and concentration flux. (**a**) At T = 0 min, prior to mixing, PTX is localized predominantly in the aqueous phase. (**b**) At T = 1 min, mixing induces directional migration of PTX molecules toward the upper extraction phase. (**c**) At T = 10 min, a clear concentration gradient is observed across the DMSO layer, with vector arrows indicating net flux predominantly toward the organic solvent. The simulation reflects the total system behavior, including mixing and settling, prior to solvent-layer separation at T = 10 min.

This equation suggests that PTX preferentially accumulates at the DMSO interface due to favorable surface energy differences, facilitating its eventual transition into the organic phase. This mechanistic understanding was incorporated into a time-dependent finite element model using COMSOL Multiphysics (the Transport of Diluted Species module). The simulation, shown in Figure 2, visualizes PTX flux across the three layers and captures the dynamics of drug migration over a 10 min extraction period.

This modeling approach highlights how interfacial behavior within a microscale extraction system can significantly influence analyte recovery and quantification. Such insights are essential for accurate interpretation of drug transport in microengineered platforms.

The computational model presented here is intended as a mechanistic and illustrative representation of drug transport during the extraction and partitioning process, rather than as a fully parameter-identified or predictive transport model. Given the limited number of experimentally constrained parameters, multiple combinations of diffusion, partitioning, and interfacial transfer coefficients could plausibly reproduce similar temporal behavior. Accordingly, the model was not used to infer unique transport parameters or to quantitatively fit experimental concentration-time data. All quantitative conclusions regarding drug uptake and absorption are derived from direct LC-MS/MS measurements. A formal sensitivity or identifiability analysis was beyond the scope of the present study.

### 3.3. Analytical Validation for High-Throughput MPS Applications

To support quantitative drug transport analysis in engineered microscale tissue systems, a linearity series was prepared in *Complete Media* to enable accurate measurement of PTX absorption across a broad concentration range. The theoretical calibration range spanned 0.02 µM to 15 µM per analytical run. However, following matrix spiking and validation across runs, 0.03 µM (L1) was identified as the lowest concentration meeting predefined accuracy and precision acceptance criteria, and was, therefore, established as the validated lower limit of quantification (LLOQ).

To ensure robustness and reproducibility, the linearity series was manufactured and validated across four independent iterations. All iterations demonstrated comparable performance across validation metrics; therefore, data from a representative iteration are reported here and were used for all subsequent experiments. Measured concentrations, assay precision (%CV), and accuracy are summarized in Table 2.

**Figure 3:**
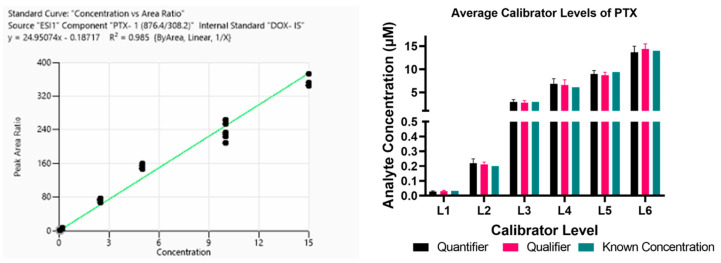
Calibration curve and validation of assay linearity. (**Left**) Representative standard curve for PTX from one linearity series run, demonstrating strong linearity with an R^2^ = 0.985. (**Right**) Average PTX concentrations measured for each calibrator level across three independent runs using the same linearity series, illustrating reproducibility and precision across the dynamic range of the assay.

The reported calibration series consisted of six concentration levels: L1 = 0.03 µM, L2 = 0.2 µM, L3 = 2.9 µM, L4 = 6.8 µM, L5 = 8.9 µM, and L6 = 13.6 µM. This series was used for all validation analyses and downstream quantification (Figure 3). Assay linearity was evaluated using the coefficient of determination (R^2^) generated for both monitored MRMs across multiple analytical runs (*n* = 3 injections per level per run; 6 runs per linearity series). The assay demonstrated robust linearity across multiple analytical runs (R^2^ ≥ 0.90), with best-run performance exceeding R^2^ = 0.99.

Assay precision was assessed using percent coefficient of variation (%CV), with an acceptance criterion of ≤20% for individual, intra-assay, and interassay measurements. Accuracy was evaluated against a target range of 100 ± 20%. Across all tested iterations, the assay consistently met both precision and accuracy requirements, confirming its suitability for high-throughput quantitative measurements in microphysiological systems.

This level of analytical validation is essential for microscale drug transport studies, where reliable quantification across time, dose, and experimental batches underpins meaningful IVIVE.

### 3.4. Validation of Assay Integrity Under Repeated Use and Storage Conditions

To ensure the assay’s suitability for high-throughput applications in microphysiological drug screening platforms, we evaluated potential carryover effects and calibrator stability under typical experimental conditions, similar to those reported in our previous studies [42,43].

**Figure 4:**
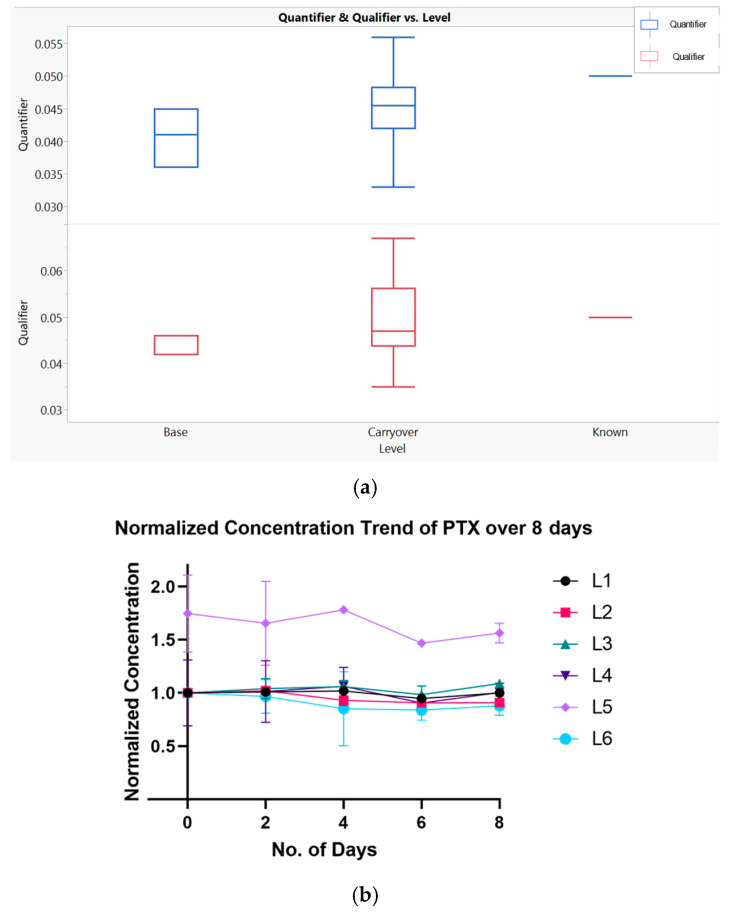
Assessment of assay carryover and calibrator stability. (**a**) Carryover analysis for the lowest calibrator level (L1) following high-concentration (L6) injections, showing the spread across sextuplet groups (*n* = 3 per group; triple injections per sample). No significant carryover was observed, with all values within ±20% of baseline. (**b**) Normalized PTX concentrations (quantifier MRM) for all calibrator levels over an 8-day stability study. Calibrators were stored at −20 °C and stressed at +4 °C, then measured in duplicate (*n* = 2 per level) with triplicate injections. All levels remained stable except L5, which showed a statistically significant deviation.

Carryover was assessed by repeatedly injecting the lowest calibration standard (L1) in triplicate immediately following the highest standard (L6), without incorporating any intermediate blank runs. This procedure was repeated across seven triplicate sets, and the results were compared to baseline L1 injections that followed two blank samples. As shown in Figure 4a, no significant outliers were observed, and the carryover remained within a ±20% threshold, meeting acceptance criteria for high-sensitivity LC–MS/MS workflows.

While PTX is known to degrade under certain in vitro conditions [17,38], its long-term stability in aqueous calibration matrices (especially for repeated LC–MS/MS analysis) remains a key consideration. To investigate this, calibration standards were prepared in duplicate and stored at −20 °C, with temperature stress applied at +4 °C over an 8-day period. Samples were retrieved at 2-day intervals, run in triplicate, and averaged for each time point.

Data was normalized to day 0 (D = 0) values for both monitored MRMs, and paired Student *t*-tests were used to evaluate statistical significance in signal drift. Results for the quantifier MRM are shown in Figure 4b (the qualifier exhibited identical trends). With the exception of calibrator L5, all levels remained stable over the 8-day study period. Notably, levels L2, L3, and L6 exhibited statistically significant (*p* < 0.05) reductions at D = 4 but remained within acceptable performance thresholds.

These findings indicate that calibrators prepared in aqueous media are sufficiently stable for up to 11 days when stored at −20 °C, enabling reliable use in repeated or delayed analysis of microscale experimental samples.

### 3.5. Drug Absorption by 3D Microtissues™

**Figure 5:**
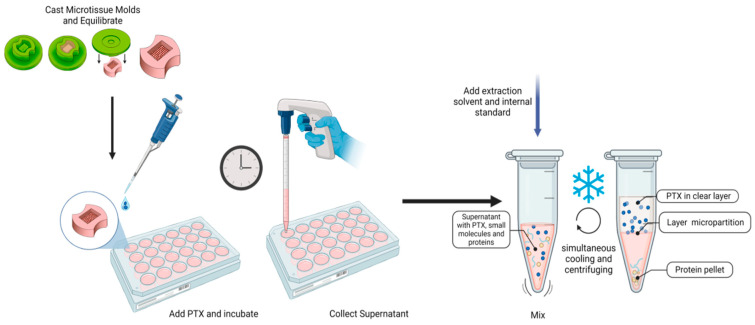
Schematic overview of the experimental workflow, including microtissue casting, drug treatment protocol, and post-treatment sample collection and phase separation for LC-MS/MS analysis. The process illustrates the scaffold-free 3D culture setup, PTX exposure, and extraction of supernatant media for quantification.

Following assay optimization and validation, PTX uptake by scaffold-free 3D *Microtissue*™ molds was evaluated across a range of treatment concentrations and time points. The experimental regimen is detailed in the *Methods* section and visually summarized in Figure 5.

**Figure 6:**
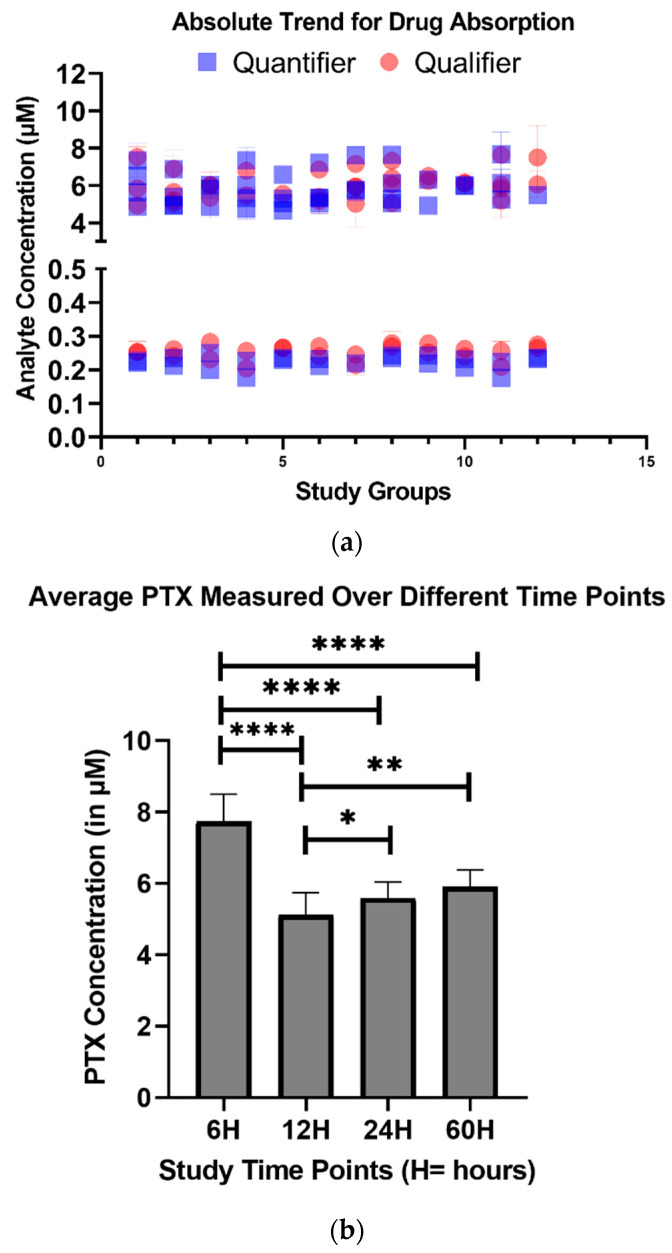
Quantitative analysis of PTX absorption by *Microtissue*™ molds. (**a**) PTX uptake trends over time for the 0.5 µM and 5 µM treatment groups, shown for both monitored MRMs. Data represent *n* = 12 microtissue molds per group. (**b**) Average intracellular PTX concentrations measured at multiple time points across all treatment conditions (*n* = 12 per time point), demonstrating statistically significant differences in drug uptake over time. Data were analyzed using Tukey–Kramer HSD post hoc testing. * *p* < 0.05, ** *p* < 0.01, **** *p* < 0.0001.

Across all treatment groups, substantial analyte absorption by the molds was observed. Specifically, for PTX doses below 1 µM, approximately 50% of the initial drug was absorbed by the *Microtissues*™ within the first 24 h. At concentrations ≥1 µM, absorption plateaued between 25 and 30%, even after 60 h of continuous exposure, as shown in Figure 6a. In contrast, control wells without the molds exhibited negligible drug loss, confirming that the observed depletion was attributable to microtissue uptake. In this study, drug absorption was quantified as net depletion of paclitaxel from the culture medium, supported by mold-free control wells that exhibited negligible drug loss under identical conditions. While this approach robustly captures platform-mediated exposure loss, it does not provide a closed mass balance across media, gel, cellular, and plastic compartments. Direct measurement of the drug retained within the mold or gel phase would further refine mechanistic interpretation but was beyond the scope of the present study.

Each condition was tested in quadruplicate across four independent experiments. For each concentration and time point, *Microtissues*™ were prepared in triplicate, and each supernatant sample was analyzed in technical triplicate (*n* = 12 per group). This level of replication enabled robust statistical comparisons across time and dose.

Time-dependent differences in drug absorption were evaluated using Tukey–Kramer HSD post hoc analysis. As shown in Figure 6b, all treatment groups exhibited statistically significant differences in absorbed PTX over time (*p* < 0.05), highlighting the dynamic nature of analyte uptake, even in non-scaffolded 3D cultures. Notably, even short exposure periods led to significant drug depletion relative to the initial treatment concentration.

These results underscore a critical consideration for microphysiological drug screening platforms: the assumed dosing concentration does not necessarily reflect the true exposure experienced by the tissue. This discrepancy introduces a potential source of error in pharmacodynamic interpretation and IVIVE models.

Furthermore, the extent of analyte absorption is likely compound-specific, influenced by factors such as molecular size, lipophilicity, and interaction with hydrogel-based microstructures. For example, Tamoxifen, a hormone-based anticancer agent with different physicochemical properties than PTX, may exhibit distinct absorption behavior even in identically prepared *Microtissue*™ molds. These findings emphasize the importance of platform-specific and molecule-specific absorption profiling as part of preclinical IVIVE workflows [44].

While this study focuses on PTX as a representative hydrophobic chemotherapeutic agent and MCF-7 breast cancer microtissues, drug absorption behavior is expected to vary substantially with molecular properties such as polarity, charge, solubility, and protein binding, as well as with platform composition and geometry. Hydrophilic or charged compounds may exhibit markedly different transport and retention profiles within the same microtissue system. Accordingly, the present work does not seek to generalize absorption behavior across all drugs or platforms, but rather to establish a transferable LC-MS/MS-based analytical framework for quantitatively characterizing platform-specific drug exposure in engineered 3D culture systems.

### 3.6. Functional Validation in Cell-Based Microtissue Constructs

To functionally validate the pharmacokinetic absorption data obtained from acellular *Microtissue*™ molds, we extended the analysis to cell-seeded constructs. MCF-7 breast cancer cells were seeded into the *Microtissue*™ molds and allowed to self-assemble into 3D spheroids prior to PTX exposure for 24 h. This experiment was performed in parallel with acellular molds to account for mold-specific drug absorption.

**Figure 7:**
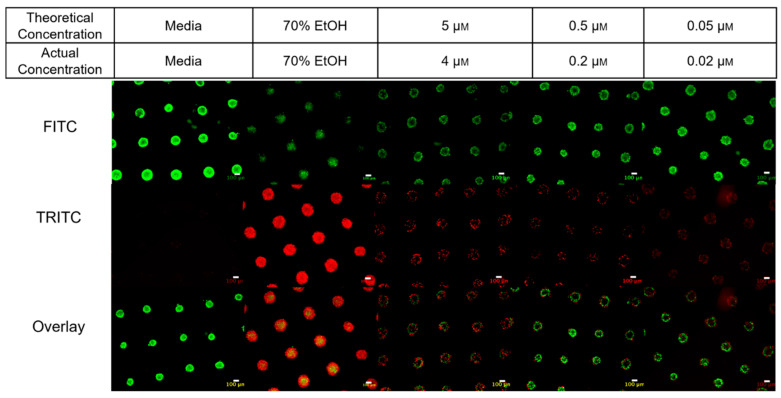
Representative fluorescence images of MCF-7 spheroids stained with live–dead assay reagents. Live cells appear green (FITC channel), and dead cells appear red (TRITC channel). Images correspond to both theoretical treatment concentrations and actual intracellular concentrations (adjusted for mold absorption). Each condition represents one section from a single microtissue mold (*n* = 3 molds per condition; representative image shown). Increased cell viability was observed in the 0.05 µM and 0.5 µM groups, consistent with lower PTX absorption and intracellular exposure at concentrations below 1 µM.

Cell viability was assessed using a live–dead fluorescence assay, while intracellular PTX concentrations were quantified via LC–MS/MS. As expected, the actual intracellular drug levels diverged significantly from nominal media concentrations, particularly at higher treatment doses. The live–dead assay results confirmed this discrepancy: lower nominal concentrations (0.05–0.5 μM) yielded predominantly viable spheroids (green fluorescence), whereas higher concentrations (5 μM) produced substantial cell death (red fluorescence), as shown in Figure 7.

Importantly, if drug absorption by the *Microtissue*™ platform had not been accounted for, these cytotoxicity results could have been misinterpreted, either overstating or understating drug efficacy depending on the dosing strategy. This highlights a key limitation of traditional 2D assays or scaffold-free 3D systems, where true analyte exposure is rarely quantified.

By integrating functional viability imaging with quantitative LC-MS/MS measurements, we demonstrate the utility of engineered microphysiological platforms that couple pharmacokinetics with biological response. This dual-validation approach enhances the interpretability of in vitro assays and supports improved extrapolation to in vivo behavior.

### 3.7. Translational Relevance and Microphysiological System Alignment

This study presents a quantitative, microscale analytical framework for drug transport analysis that integrates:

- Engineering-grade, scaffold-free 3D *Microtissues*™ constructs;
- LC-MS/MS-based drug quantification;
- Finite-element modeling of interfacial and extraction-phase transport;
- Functional cytotoxicity validation using live–dead assays.

Within this workflow, the *Microtissues*™ platform functions as a microscale drug transport assessment system, enabling time- and dose-dependent evaluation of drug uptake and retention under controlled in vitro conditions.

This approach aligns with the objectives of Organs-on-a-Chip and microphysiological systems (MPS) research, where accurate IVIVE depends on quantitative characterization of drug bioavailability within engineered microenvironments. Importantly, these findings demonstrate that scaffold-free platforms are not exempt from drug absorption effects and that platform-specific drug sequestration must be accounted for when interpreting pharmacological responses.

By integrating direct molecular quantification with phenotypic response, this framework advances mechanistically informed evaluations of engineered microphysiological platforms, supporting improved interpretability of 3D drug screening data.

Future work will extend this framework to patient-derived microtissues and perfusion-enabled microfluidic systems, increasing physiological relevance and translational utility for oncology and toxicity assessment.

## 4. Conclusions

IVIVE remains central to translating preclinical findings into clinically meaningful insights, particularly as engineered microscale tissue systems increasingly replace traditional 2D culture models. Achieving reliable IVIVE requires direct and quantitative determination of drug exposure within these platforms rather than reliance on nominal dosing assumptions.

In this study, we established a validated LC-MS/MS-based analytical framework for quantifying paclitaxel absorption and exposure dynamics in scaffold-free 3D *Microtissues*™. Using optimized liquid–liquid extraction and rigorously validated mass spectrometric assays, we demonstrate that platform-specific drug sequestration can substantially alter true intracellular exposure, even in nonadhesive hydrogel-based constructs. Complementary computational modeling was employed to support the mechanistic interpretation of transport and partitioning behavior but was not used for parameter inference.

These findings position LC-MS/MS as a critical enabling technology for quantitative characterization of microphysiological systems. Incorporating direct analyte measurement into 3D culture workflows is essential for accurate interpretation of dose–response relationships and for improving the reliability of downstream IVIVE analyses.

As biofabricated microscale platforms continue to evolve, rigorous mass-spectrometry-based quantification of drug–tissue interactions will be essential for advancing predictive drug screening, translational bioengineering, and precision oncology research.

## Figures and Tables

**Table 1:** (**a**) Optimized MS parameters for PTX and DOC (IS) on the QSight; (**b**) LC gradient flow design for the elution of PTX and DOC.

(**a**)					
	**Q1 Mass**	**Q3 Mass**	**EV**	**CC**	**CCL2**
Paclitaxel—Quantifier	876.4	308.2	32	−48	−376
Paclitaxel—Qualifier	876.4	531.2	41	−30	−210
Docetaxel—IS	830.4	549.1	33	−34	−209
(**b**)					
**Time (min)**			**Flow Rate**	**% A**	**% B**
0			0.4	95	5
0.25			0.4	37	63
0.9			0.4	27	73
1			0.4	5	95
1.5			0.4	5	95
1.51			0.4	27	73
2			0.4	37	63
3			0.4	95	5

**Table 2:** (**a**) Quantifier; (**b**) qualifier. Average concentrations, standard deviation, %CV and % accuracy for PTX calibrators (*n* = 3 per level, per run; total number of runs = 6).

(**a**)					
Calibrator Level	$\mu_{\bar{x}}\pm \sigma$	%CV	% Accuracy	Interassay %CV	Intra-Assay %CV
L1	0.03±0.01	17.8%	83%	16.7%	8.9%
L2	0.22±0.03	13.2%	110%		
L3	2.93±0.50	17.0%	101%		
L4	6.82±1.3	16.6%	111%		
L5	8.97±0.76	8.4%	95%		
L6	13.66±1.34	9.8%	97%		
(**b**)					
Calibrator Level	$\mu_{\bar{x}}\pm \sigma$	%CV	%Accuracy	Interassay %CV	Intra-Assay %CV
L1	0.03±0.01	17.2%	102%	12.5%	8.2%
L2	0.21±0.01	6.91%	96%		
L3	2.80±0.42	15.2%	97%		
L4	6.56±1.2	17.5%	109%		
L5	8.72±0.62	7.2%	92%		
L6	14.40±1.07	7.4%	101%		

## Data Availability

Additional data can be made available upon reasonable request from the authors.

## Supplementary Materials

The following supporting information can be downloaded at: https://www.mdpi.com/article/10.3390/mi17030332/s1, Table S1. Comparison between 2D and 3D cell culture models (compiled from literature) [12,13,14]. Table S2. The advantages and disadvantages of existing 3D cell culture techniques against *Microtissues* [3,10,18,19]. Figure S1. MS spectra showing the mass fragments identified and used for this study. Figure S2. Closed in vitro system of PTX in *Complete Media* penetrating and entering the *Microtissue* mold, while equilibration media diffuses out of the mold to the surroundings. PTX has to overcome the mold barrier to finally reach the spheroids, and depending on the time of the study, some media evaporation also occurs.
