# Supplementary material for: Integrated Experimental–Computational Framework for Drug Transport Quantification in 3D Microtissues™
Source: Micromachines (Basel). 2026 Mar 9;17(3):332. doi: 10.3390/mi17030332 (PMC13028606; doi:10.3390/mi17030332)
Supplement: Supplementary file 1 [file micromachines-17-00332-s001.zip › micromachines-4151835-supplementary.pdf]

Integrated Experimental-Computational Framework for Drug Transport Quantification in 3D Microtissues™

Ramisa Fariha[1], Jad Hamze[2], Oluwanifemi David Okoh[1], Emma Rothkopf[1], Anubhav Tripathi[1*]

1. Center for Biomedical Engineering, School of Engineering, Brown University, 182 Hope Street, Providence, RI- 02912, USA
2. Department of Biology and Medicine, Brown University, Providence, RI- 02912, USA

* Corresponding Author: anubhav_tripathi@brown.edu

Supplemental Information

	Characteristics	2D Cell Culture	3D Cell Culture
1	Cell Morphology	Cultures deform cell shape due to growth on a flat 2D surface	Actual shape is maintained with spheroid, organoid and other 3D cell culturing models
2	Cell Proliferation	Growth of cells is too rapid and therefore is unable to mimic that of in vivo growth	Growth of cells under 3D conditions is better able to replicate actual cell growth in vivo
3	Cell and ECM proliferations	Growth on a flat surface is not an appropriate replication of the actual environment of a tissue due to lack of ECM and cell interactions.	Due to the 3-dimensional structure, cells and ECM are able to interact replicating what occurs in vivo.
4	Cell-Cell Interactions	The multi-cells interactions occurring are unable to accurately depict what occurs in native organ environment	Multi-cell interactions in varied 3D cell culturing models are able to replicate native environments
5	Cell Differentiation	Unable to accurately mimic native tissue	Better resemblance of differentiation in native tissue with markers expression more similar to native tissue
6	Drug Response efficacy	Unable to see proper drug efficacy due to plastic substrate environment that cells are maintained within	More predictable due to the similarity's due in vivo environment
7	Apoptosis and viability (tumor models)	More sensitive relative to what occurs in vivo when studying target drugs	Higher resistance to anti-cancer target drugs, better resembling what occurs in vivo.
8	Physiological relevance	Not relevant due to lack of recapitulation of tumor microenvironment	Feasible to make physiologically relevant nutritional and oxygen conditions
9	Experimentation and analysis	Simple to use and very replicable data	Relative to 2-dimensional models, they are more complicated to use and harder to replicate
10	Characterizations	Ease of characterization regardless of instrument. Further, techniques are reviewed and readily available	More difficult to characterize due to the necessity of certain instrumentation as well as more time-consuming
11	Cost	More well-established and relatively inexpensive	More expensive and less standardization due to lack of studies.

Supplemental Table S1. *Comparison between 2D and 3D cell culture models (compiled from literature) [12-14].*

	**Microtissues**	**Spontaneous Aggregation**	**Liquid Overlay Cultures**	**Scaffold based cultures**	**Gyratory and Spinner Flasks**	**Pre-engineered collagen composite scaffolds**	**Rotary Cell Culture System**
**Cost**	Relatively inexpensive	Inexpensive	Inexpensive	Expensive	Inexpensive	Expensive	Expensive
**Ease of Use**	Easy to prepare and use	Easy to prepare and use	Easy to prepare and use	Easy to prepare and use	Easy to prepare and use	Easy to prepare and use	Easy to prepare and use (quick)
**Spheroid Rigidity**	Well-defined controlled spheroids	Cluster and not well-defined spheroids	Concave bottom results in defined spherical shape	well-defined controlled spheroids	uncontrollable size and not well-defined spheroids	forms well-defined sepheroids with temperature alteration	well-defined controlled spheroids
**Through put Ability**	High	Relatively Low	Low	Low	Relatively Low	High	High
**ECM Support**	Scaffold-Free	Successfully mimics in vivo ECM	High when Hyaluronan present	Cells grow on thick layer of ECM resulting in cell differentiation	Use natural and synthetic ECM mimicking hydrogels	Mimics in vivo ECM well	Mimics in vivo ECM well

Supplemental Table S2. *The advantages and disadvantages of existing 3D cell culture techniques against Microtissues [3,10,18,19].*

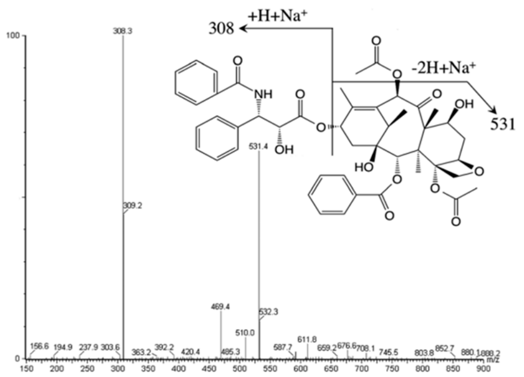

Supplemental Figure S1. MS spectra showing the mass fragments identified and used for this study.

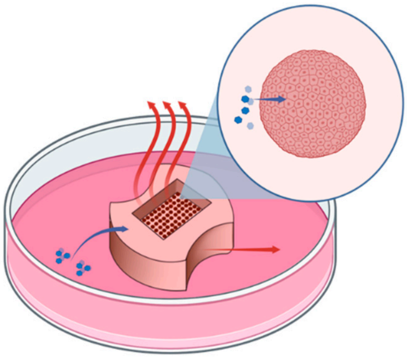

Supplemental Figure S2. Closed in vitro system of PTX in Complete Media penetrating and entering the Microtissue mold, while equilibration media diffuses out of the mold to the surroundings. PTX has to overcome the mold barrier to finally reach the spheroids, and depending on the time of the study, some media evaporation also occurs.
